# Homeostatic Presynaptic Plasticity Is Specifically Regulated by P/Q-type Ca^2+^ Channels at Mammalian Hippocampal Synapses

**DOI:** 10.1016/j.celrep.2017.09.061

**Published:** 2017-10-10

**Authors:** Alexander F. Jeans, Fran C. van Heusden, Bashayer Al-Mubarak, Zahid Padamsey, Nigel J. Emptage

**Affiliations:** 1 Department of Pharmacology, University of Oxford, Mansfield Road, Oxford OX1 3QT, UK; 2 Department of Genetics, King Faisal Specialist Hospital and Research Center, PO Box 3354, Riyadh 11211, Saudi Arabia

**Keywords:** synapse, homeostatic plasticity, voltage-gated calcium channel, neurotransmitter release, pHluorin, P/Q-type channelopathy

## Abstract

Voltage-dependent Ca^2+^ channels (VGCC) represent the principal source of Ca^2+^ ions driving evoked neurotransmitter release at presynaptic boutons. In mammals, presynaptic Ca^2+^ influx is mediated mainly via P/Q-type and N-type VGCC, which differ in their properties. Changes in their relative contributions tune neurotransmission both during development and in Hebbian plasticity. However, whether this represents a functional motif also present in other forms of activity-dependent regulation is unknown. Here, we study the role of VGCC in homeostatic plasticity (HSP) in mammalian hippocampal neurons using optical techniques. We find that changes in evoked Ca^2+^ currents specifically through P/Q-type, but not N-type, VGCC mediate bidirectional homeostatic regulation of both neurotransmitter release efficacy and the size of the major synaptic vesicle pools. Selective dependence of HSP on P/Q-type VGCC in mammalian terminals has important implications for phenotypes associated with P/Q-type channelopathies, including migraine and epilepsy.

## Highlights

• P/Q-type VGCC regulate homeostatic synaptic plasticity (HSP) in mammals
• Changes in synaptic vesicle pool sizes during HSP are also mediated via P/Q-type VGCC
• Expression of HSP is independent of N-type VGCC regulation
• P/Q-type regulation of HSP may explain phenotypes of P/Q-type channelopathies

## Graphical Abstract

**Figure:**
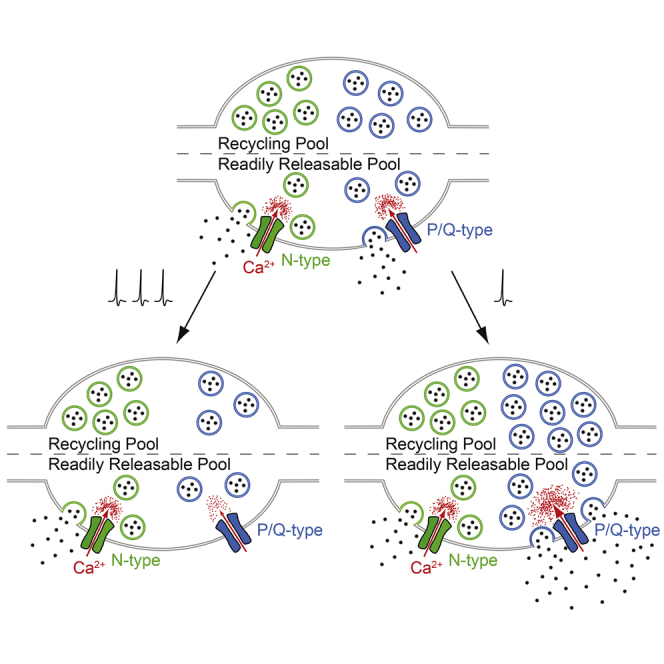

## Teaser

Jeans at al. show that both basal neurotransmission and synaptic vesicle pool sizes are specifically regulated by the presynaptic P/Q-type voltage-gated Ca^2+^ channel during HSP at mammalian hippocampal synapses. This may shed light on mechanisms underlying phenotypes associated with P/Q-type channelopathies, including migraine and epilepsy.

## Introduction

Voltage-gated Ca^2+^ channels (VGCC) provide the essential link between membrane depolarization and synaptic vesicle exocytosis at presynaptic boutons and play a critical role in tuning neurotransmission (Catterall and Few, 2008). In the mammalian hippocampus, P/Q-type (Ca_v_2.1) and N-type (Ca_v_2.2) VGCC are the main presynaptic sources of Ca^2+^ influx (Takahashi and Momiyama, 1993, Wheeler et al., 1994). The proportional contribution of P/Q- and N-type VGCC to neurotransmitter release varies among individual synapses (Ariel et al., 2013) and, given their differing functional properties (Catterall and Few, 2008), decisively shapes synaptic transmission both during development and in Hebbian plasticity (Ahmed and Siegelbaum, 2009, Fedchyshyn and Wang, 2005). However, whether the differential control of neurotransmission by VGCC represents a general principle that extends to other forms of activity-dependent synaptic regulation is not known.

Homeostatic synaptic plasticity (HSP) represents a major form of synaptic plasticity that is essential for maintaining stability and efficient information processing in neuronal networks in the face of constantly changing levels of activity (Turrigiano, 2012). Both pre- and postsynaptic changes mediate HSP, although the contribution of each seems to be dependent on several factors including age: at younger cortical and hippocampal synapses, for instance, HSP is exclusively postsynaptic, but a presynaptic locus of expression emerges as circuits mature (Han and Stevens, 2009, Wierenga et al., 2005, Wierenga et al., 2006). Presynaptic HSP has been best studied at the *Drosophila* neuromuscular junction, where homeostatic adjustments in neurotransmitter release are mediated by a signaling system that converges on P/Q-type VGCC encoded by the locus *cacophony* (Davis and Müller, 2015). However, evoked neurotransmitter release in *Drosophila* is driven by *cacophony*-encoded VGCC alone (Littleton and Ganetzky, 2000), whereas the N-type VGCC seems to have arisen later in evolution as the result of a gene duplication event (Tyson and Snutch, 2013). The combinatorial regulation of release by presynaptic VGCC seen at mammalian synapses is not, therefore, a feature of this system. In mammals, N-type, but not P/Q-type, VGCC function is regulated by the enzyme CDK5 (Kim and Ryan, 2013, Su et al., 2012), a key effector of HSP both pre- and postsynaptically (Kim and Ryan, 2010, Seeburg et al., 2008). This has led to an assumption that N-type channels are likely to be the sole presynaptic VGCC mediating HSP at mammalian synapses (Frank, 2014, Kim and Ryan, 2013). However, the regulation of release by VGCC has never been directly investigated in the context of the chronic changes in network activity necessary to induce HSP (Turrigiano et al., 1998).

We set out to better understand how VGCC support HSP at mammalian synapses using high-resolution optical methods to probe both Ca^2+^ influx and neurotransmitter release in dissociated hippocampal cultures. We find that changes in evoked Ca^2+^ currents specifically through P/Q-type channels support bidirectional homeostatic changes in both the efficacy of neurotransmitter release and the size of the major synaptic vesicle pools. Our findings suggest that the differential regulation of transmitter release by VGCC subtypes may be a general principle by which presynaptic function is regulated in different activity-dependent contexts and have intriguing implications for the pathogenesis of key phenotypes associated with P/Q-type Ca^2+^ channelopathies, including migraine and epilepsy.

## Results

### Homeostatic Changes in Presynaptic Ca^2+^ Influx and Glutamate Release

**Figure 1:**
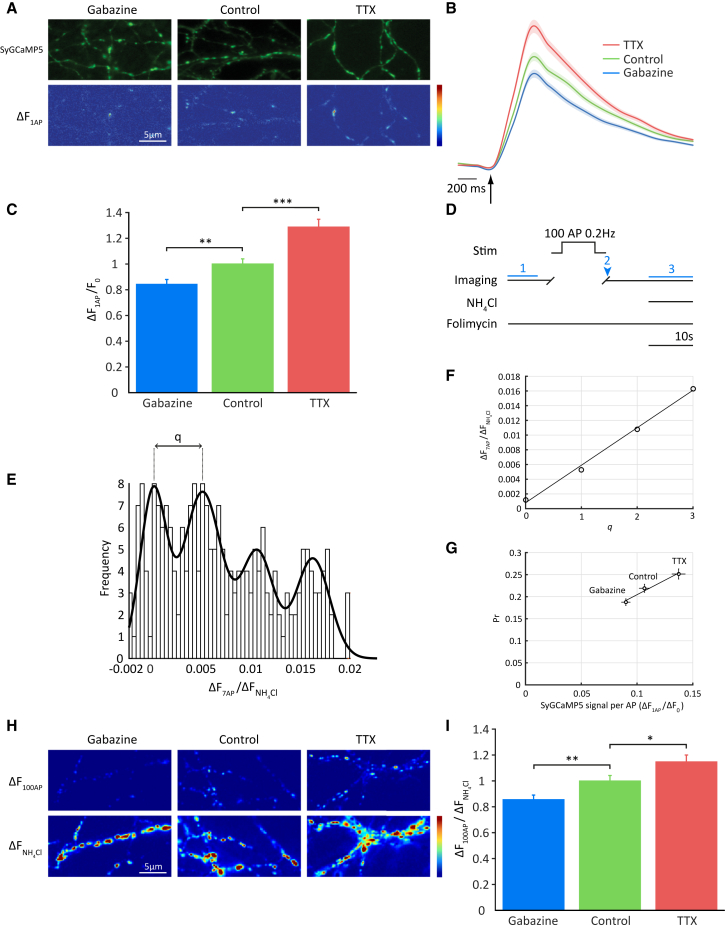
Homeostatic Changes in Presynaptic Ca^2+^ Influx and Neurotransmitter Release at Mammalian Synapses (A) Sample images showing rat hippocampal neurons expressing the presynaptic Ca^2+^ indicator SyGCaMP5 and subjected to chronic drug treatments as indicated. Difference images in the lower panels show responses of terminals to a single AP. (B) Average SyGCaMP5 fluorescence traces obtained in response to the 1 AP stimulus. Arrow denotes delivery of stimulus. (C) Mean peak amplitudes of normalized Ca^2+^ responses: gabazine treatment: 0.843 ± 0.037 (n = 584 synapses from 17 cells); vehicle-treated control, 1 ± 0.040 (n = 695 synapses from 18 cells); TTX treatment, 1.286 ± 0.061 (n = 479 synapses from 14 cells). (D) Experimental scheme for determination of summated SypH 2x pHluorin responses to single AP stimuli: cells are imaged before (1) and after (2) a period of low-frequency stimulation (100 AP/0.2 Hz) in the presence of the V-ATPase inhibitor folimycin, which prevents synaptic vesicle re-acidification. The signal change is normalized to the maximally unquenched signal (3) to adjust for pHluorin expression level differences between cells. (E) Release in response to a 0.2 Hz stimulus train is quantal. Following a 7 AP/0.2 Hz stimulus in the presence of folimycin, a frequency distribution of responses was plotted (n = 526 synapses from 11 cells). The optimized Gaussian fit comprises 4 equally spaced distributions. (F) Calculation of the interpeak distance using linear regression allows an estimate of quantal size (*q*) as the slope of a plot of the mean of each Gaussian (q = 0.005 ΔF/F_NH4Cl_). Pr was then calculated from the 100 AP response in a sample of control neurons as 0.219 ± 0.009 (n = 174 synapses from 7 cells), in excellent agreement with previous reports. (G) This method is sensitive enough to detect changes in basal Pr of <10% in response to the relatively small changes in presynaptic Ca^2+^ influx accompanying homeostatic regulation (data derived from I). (H) Sample images showing response of SypH 2x-expressing terminals treated as indicated to a low-frequency 100 AP train in the presence of folimycin. (I) Mean normalized SypH 2x responses: gabazine treatment: 0.856 ± 0.0346 (n = 180 synapses from 7 cells); vehicle-treated control, 1 ± 0.042 (n = 174 synapses from 7 cells); TTX treatment, 1.148 ± 0.052 (n = 214 synapses from 5 cells). Shading or error bars represent ± SEM. ^∗^p < 0.05, ^∗∗^p < 0.01, ^∗∗∗^p < 0.0001.

To study the homeostatic effect of chronic changes in network activity on presynaptic calcium influx, we imaged cultured hippocampal neurons expressing SyGCaMP5, a genetically encoded Ca^2+^ reporter localized to synaptic vesicles (Akerboom et al., 2012). Neurons were pretreated for 24–36 hr with either the Na^+^ channel blocker TTX, to block spiking activity, or the GABA_A_ receptor antagonist gabazine, to depress inhibitory tone, and the drugs were washed out before responses to single action potential (AP) stimuli were measured (Figure 1A). As expected, TTX-treated neurons showed a larger averaged single AP SyGCaMP5 response than did vehicle-treated control neurons, while the responses of gabazine-treated neurons were reduced (Figures 1B and 1C). We confirmed that our stimulation protocol reliably elicits single AP and that these drive presynaptic Ca^2+^ influx that generates a detectable SyGCaMP5 signal (Figure S1).

To explore homeostatic regulation of glutamate release, we took advantage of SypH 2x, a genetically encoded reporter of synaptic vesicle fusion (Zhu et al., 2009). Synaptic pHluorin probes have previously been used to detect responses to single AP stimulation, albeit with multiple trial averaging (Kim and Ryan, 2013, Zhao et al., 2011); however, we planned to use VGCC blocking peptides in many of our experiments, which drastically impair evoked release (Ariel et al., 2013). Indeed, preliminary data suggested that incubation with these peptides lowers averaged single AP responses below the threshold for detection in a substantial fraction of boutons (data not shown). Therefore, we needed to develop an approach to detect single AP release events under conditions of low release efficacy with enhanced sensitivity. To do this, we delivered 100 AP stimulation at very low frequency (0.2 Hz) in the presence of the vesicular ATPase inhibitor folimycin (Figure 1D), which blocks the reacidification of synaptic vesicles so that the pHluorin signal represents cumulative exocytosis (Sankaranarayanan and Ryan, 2001). This stimulation protocol induces repetitive release with little or no short-term plasticity (Tokuoka and Goda, 2008), allowing effective summation of single AP responses over 100 trials to give a greatly improved signal-to-noise ratio. To confirm that low frequency stimulation yields quantal responses, we delivered a shorter, 7 AP train of stimuli at the same frequency in folimycin and plotted the distribution of the signal change, background-adjusted and normalized to the maximal value revealed by unquenching with NH_4_Cl, across terminals (Tokuoka and Goda, 2008). The optimal Gaussian fit was obtained with 4 equally spaced distributions, confirming the quantal nature of the response (Figure 1E). The interpeak distance, which represents quantal size (q), could then be calculated using linear regression (Figure 1F). To further validate our method, we used this estimate of q to calculate the probability of release (Pr) of control terminals in culture. Taking the 100 AP SypH 2x response, ΔF_100AP_, Pr was calculated as (ΔF_100AP_/100)/*q*. Average Pr in control cultures determined by this method (0.219 ± 0.009, n = 174 synapses) was very similar to that previously reported (Tokuoka and Goda, 2008). Finally, we proved the sensitivity of our method by demonstrating its ability to detect unambiguously Pr changes in the region of 10% in response to the relatively subtle changes in presynaptic Ca^2+^ influx associated with homeostatic regulation (Figure 1G). Specifically, we found that, as for presynaptic Ca^2+^ influx, pretreatment with TTX enhanced glutamate release while it was reduced following gabazine treatment. These results show that chronic changes in network activity elicit parallel changes in both presynaptic Ca^2+^ influx and release, consistent with previous findings (Zhao et al., 2011).

### Mechanisms of Homeostatic Change in Presynaptic Ca^2+^ Influx

**Figure 2:**
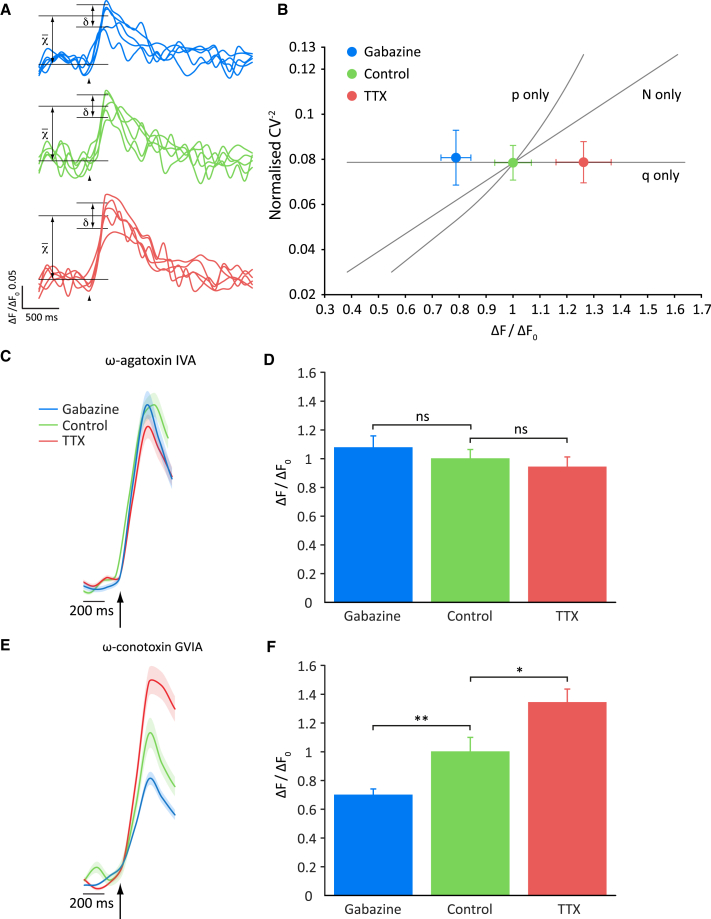
P/Q-type VGCC Drive Homeostatic Changes in Presynaptic Ca^2+^ Influx (A) Optical fluctuation analysis of Ca^2+^ responses: fluctuation of responses in gabazine-treated, control, and TTX-treated cells using data from Figure 1C ($\bar{\chi }$, mean; δ, SD of Δ[Ca^2+^]; arrowheads indicate delivery of AP). (B) Relationship between the mean of the inverse squared coefficient of variation (CV^−2^) and mean amplitude of response in each of the three treatment conditions. The lines are predictions of what would be observed if homeostatic changes in Ca^2+^ response amplitude were explained solely by changes in the number of VGCC (N), their open probability (p) or free Ca^2+^ concentration per channel opening (q). Data did not significantly deviate from what is predicted for changes in q (p = 0.59), but showed very significant deviation from predictions for N or p change (both p < 0.01). (C) Average SyGCaMP5 fluorescence traces obtained in response to the 1AP stimulus in the presence of ω-agatoxin IVA. Arrow represents delivery of AP. (D) Mean peak amplitudes of normalized responses in ω-agatoxin IVA: gabazine treatment, 1.077 ± 0.082 (n = 233 synapses from 6 cells); vehicle-treated control, 1 ± 0.064 (n = 269 synapses from 8 cells); TTX treatment, 0.942 ± 0.042 (n = 206 synapses from 8 cells). (E) Average SyGCaMP5 fluorescence traces obtained in response to the 1 AP stimulus in the presence of ω-conotoxin GVIA. Arrow represents delivery of AP. (F) Mean peak amplitudes of normalized responses in ω-conotoxin GVIA: gabazine treatment: 0.699 ± 0.042 (n = 414 synapses from 16 cells); vehicle-treated control, 1 ± 0.10 (n = 310 synapses from 10 cells); TTX treatment, 1.343 ± 0.094 (n = 295 synapses from 13 cells). Shading or error bars represent ± SEM. ^∗^p < 0.05, ^∗∗^p < 0.01; ns, non-significant.

We first asked whether changes in number or function of presynaptic VGCC underlie the homeostatic changes in Ca^2+^ influx. Optical fluctuation analysis uses calculations of the coefficient of variation (CV) of Ca^2+^ responses over individual trials to distinguish whether alterations in the magnitude of the Ca^2+^ response are due to changes in the number of VGCC present in presynaptic terminals (N), their probability of opening in response to an AP (p), or in intraboutonal free Ca^2+^ per channel opening (q), which typically represents unitary channel conductance (Sabatini and Svoboda, 2000). Following either gabazine or TTX treatment, the CV^−2^ of Ca^2+^ responses was unchanged relative to untreated controls despite changes in mean response amplitude (Figures 2A and 2B), which is consistent with a modulation of q underlying bidirectional homeostatic changes in Ca^2+^ responses without change in N or p.

Neurotransmitter release at mammalian CNS synapses is driven by a combination of P/Q-type and N-type VGCC (Takahashi and Momiyama, 1993, Wheeler et al., 1994). To determine the role played by each in mediating homeostatic changes in presynaptic Ca^2+^ influx, SyGCaMP5-expressing neurons were treated for 24–36 hr with TTX, vehicle, or gabazine before the drugs were washed out and responses to single AP stimulation were monitored in the presence of highly specific peptide blockers of either P/Q-type (ω-agatoxin IVA) or N-type (ω-conotoxin GVIA) VGCC. Agatoxin completely abolished the effect of chronic manipulation of activity on presynaptic Ca^2+^ influx (Figures 2C and 2D), whereas the differences between the drug-treated groups were retained in the presence of conotoxin (Figures 2E and 2F). These data indicate that P/Q-type, but not N-type, VGCCs are required for presynaptic homeostatic effects. Overall, our results thus far suggest that modulation of P/Q-type VGCC unitary conductance underlies bidirectional homeostatic changes in presynaptic Ca^2+^ influx in response to chronic alterations in network activity.

### Mechanisms of Homeostatic Change in Synaptic Vesicle Exocytosis

**Figure 3:**
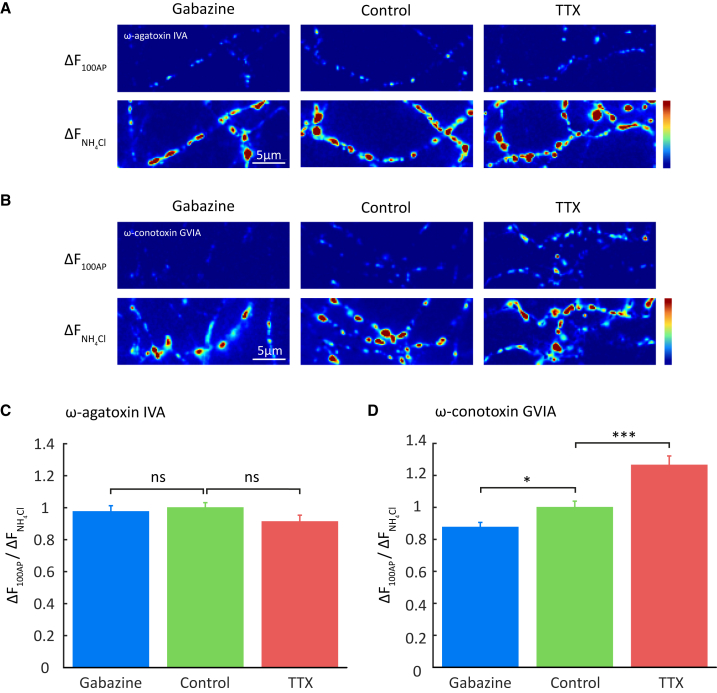
Homeostatic Changes in Neurotransmitter Release Are Driven by P/Q-type VGCC (A and B) Sample images showing response of SypH 2x-expressing terminals treated as indicated to a low-frequency 100 AP train in the presence of folimycin and either ω-agatoxin IVA (A) or ω-conotoxin GVIA (B). (C) Mean normalized responses in ω-agatoxin IVA: gabazine treatment, 0.975 ± 0.037 (n = 333 synapses from 15 cells); vehicle-treated control, 1 ± 0.031 (n = 416 synapses from 19 cells); TTX treatment, 0.912 ± 0.041 (n = 354 synapses from 17 cells). (D) Mean normalized responses in ω-conotoxin GVIA: gabazine treatment: 0.876 ± 0.045 (n = 377 synapses from 11 cells); vehicle-treated control, 1 ± 0.039 (n = 375 synapses from 14 cells); TTX treatment, 1.264 ± 0.057 (n = 282 synapses from 12 cells). Shading or error bars represent ± SEM. ^∗^p < 0.05, ^∗∗∗^p < 0.001; ns, non-significant.

As VGCC-mediated presynaptic Ca^2+^ influx is the major source of calcium for AP-dependent neurotransmitter release (Catterall and Few, 2008), we next sought to confirm whether the selective requirement of P/Q-type channels for homeostatic changes in presynaptic calcium influx is also true for neurotransmitter release. Chronically treated SypH 2x-expressing neurons were again imaged before and after delivery of a low frequency 100 AP stimulus in folimycin, this time with either agatoxin or conotoxin added. In agreement with our SyGCaMP5 data, we found that agatoxin application abolished homeostatic changes in release induced by the chronic drug treatments (Figures 3A and 3C), whereas the effects of homeostasis were fully apparent in conotoxin (Figures 3B and 3D).

**Figure 4:**
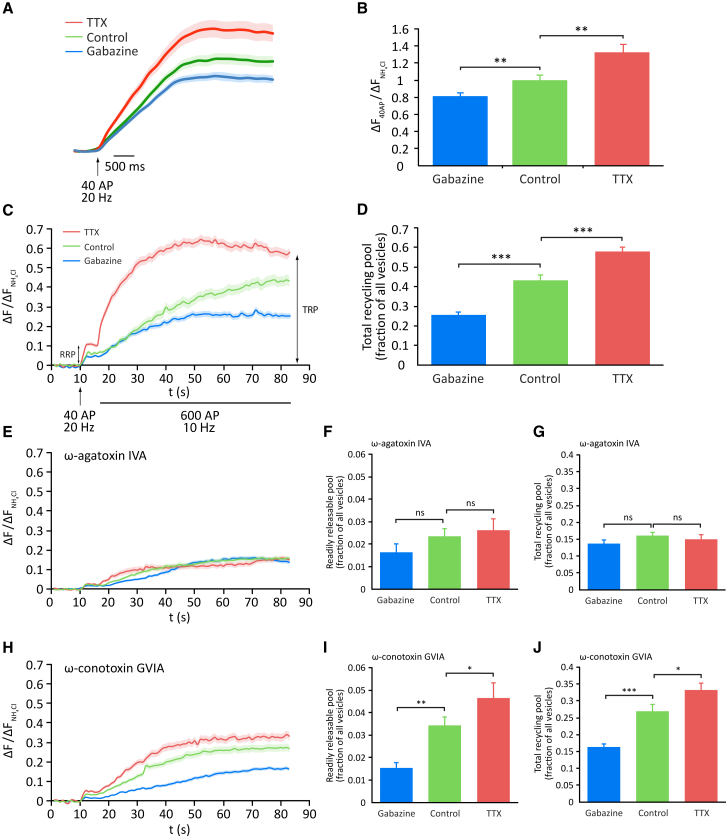
HSP Elicits Bidirectional Changes in Readily Releasable and Total Recycling Pool Size Driven by P/Q-type VGCC (A) Average SypH 2x traces showing responses to 40 AP at 20 Hz, a stimulus that depletes the readily releasable pool (RRP) of synaptic vesicles. (B) Mean peak amplitudes of 40 AP responses: gabazine treatment, 0.814 ± 0.0429 (n = 327 synapses from 10 neurons); control, 1 ± 0.0590 (n = 208 synapses from 7 neurons); TTX treatment, 1.326 ± 0.0940 (n = 250 synapses from 10 neurons). (C) Average SypH 2x responses to a stimulation protocol comprising an initial 40 AP at 20 Hz, to release the RRP, followed by 600 AP at 10 Hz to release the remainder of the total recycling pool (TRP) under control conditions and following induction of HSP with chronic drug treatments as indicated. Experiments include folimycin, therefore the fluorescence signal represents a cumulative response. (D) Mean peak amplitudes of final plateau responses, representing TRP: gabazine treatment, 0.256 ± 0.014 (n = 82 synapses from 3 cells); vehicle-treated control, 0.432 ± 0.026 (n = 97 synapses from 3 cells); TTX treatment, 0.579 ± 0.022 (n = 93 synapses from 3 cells). (E) Average SypH 2x traces for experiments performed as (C) with the addition of ω-agatoxin IVA to isolate N-type VGCC. (F) Mean peak amplitude of the initial 40 AP response in agatoxin, representing the RRP: gabazine treatment, 0.0164 ± 0.0036 (n = 63 synapses from 3 cells); vehicle-treated control, 0.0236 ± 0.0032 (n = 123 synapses from 3 cells); TTX treatment, 0.0262 ± 0.0050 (n = 93 synapses from 3 cells). (G) Final plateau response in agatoxin, representing the TRP: gabazine treatment, 0.137 ± 0.010 (n = 63 synapses from 3 cells); vehicle-treated control, 0.161 ± 0.0097 (n = 123 synapses from 3 cells); TTX treatment, 0.150 ± 0.014 (n = 93 synapses from 3 cells). (H) Average SypH 2x traces for experiments as (C) with the addition of ω-conotoxin GVIA to isolate P/Q-type VGCC. (I) Mean peak amplitude of the initial 40 AP response in conotoxin, representing the RRP: gabazine treatment, 0.0153 ± 0.0024 (n = 78 synapses from 3 cells); vehicle-treated control, 0.0344 ± 0.0037 (n = 121 synapses from 3 cells); TTX treatment, 0.0464 ± 0.0068 (n = 86 synapses from 3 cells). (J) Final plateau response in conotoxin, representing the TRP: gabazine treatment, 0.163 ± 0.0093 (n = 78 synapses from 3 cells); vehicle-treated control, 0.270 ± 0.019 (n = 121 synapses from 3 cells); TTX treatment, 0.332 ± 0.020 (n = 86 synapses from 3 cells). Shading or error bars represent ± SEM. ^∗^p < 0.05, ^∗∗^p < 0.01, ^∗∗∗^p < 0.001; ns, non-significant.

Homeostatic changes in neurotransmitter release could involve changes in addition to altered presynaptic Ca^2+^ influx. Measurements of basal presynaptic strength can be parsed into two parameters: the number of synaptic vesicles available for release upon arrival of a single AP, known as the readily releasable pool (RRP), and the probability that a vesicle in the RRP will undergo AP-evoked fusion (Schneggenburger et al., 2002), the latter a function of both presynaptic Ca^2+^ influx and the physical coupling of VGCC to release sites (Catterall and Few, 2008). To assess VGCC-release site coupling in HSP, we examined the effects of pre-incubation with the slow Ca^2+^ buffer EGTA-AM (200 μM for 60 s) on release in neurons in which P/Q-type VGCC had been isolated by the addition of ω-conotoxin GVIA (Müller and Davis, 2012). We found that induction of HSP had no effect on the EGTA sensitivity of release, indicating that coupling is unchanged (Figure S2). To probe possible changes in the size of the RRP during HSP, we stimulated neurons expressing SypH 2x with 40 AP at 20 Hz, a protocol widely used to fully deplete the RRP (Rosenmund and Stevens, 1996). Both gabazine and TTX-treated neurons showed significant differences to controls (Figures 4A and 4B), suggesting bidirectional regulation of RRP size in homeostasis.

Given our previous findings, we wondered whether changes in RRP size might be mediated via a specific VGCC subtype. We wished, in addition, to extend our studies to include the total recycling pool (TRP), the entire pool of vesicles that can be released during synaptic activity. The TRP plays a critical role in determining the efficacy of synaptic function during prolonged stimulation and has been shown to be enhanced by chronic silencing of network activity (Kim and Ryan, 2010). Two successive stimulus trains, an initial 40 AP at 20 Hz followed by 600 AP at 10 Hz, were delivered to chronically treated SypH 2x-expressing neurons in the presence of folimycin in order to deplete the RRP and TRP, respectively (Fernandez-Alfonso and Ryan, 2008). We found that, as for the RRP, the overall size of the TRP is bidirectionally regulated by HSP (Figures 4C and 4D). When we then carried out the same experiments in either agatoxin or conotoxin to isolate N- or P/Q-type VGCC, we found that plateau signals following both stimulus trains were superimposed under all conditions in agatoxin (Figures 4E–4G), but in conotoxin showed very clear bidirectional regulation by HSP in a manner that reflected changes in overall RRP and TRP size (Figures 4H–4J). For both RRP and TRP under all conditions, the sum of the pool accessible through N- or P/Q-type VGCC alone was approximately equal to the total size of the pool measured without VGCC blockade. Together, these results show that HSP elicits changes in both basal release efficacy and the size of the major functional synaptic vesicle pools that are mediated specifically through P/Q-type, and not N-type, VGCC.

## Discussion

We have used an optical approach to examine the role of VGCC in synaptic homeostasis at mammalian terminals, finding that homeostatic changes in evoked presynaptic Ca^2+^ currents, basal neurotransmitter release efficacy, and the size of the major functional synaptic vesicle pools are mediated specifically via P/Q-type VGCC.

Using optical fluctuation analysis, we showed that homeostatic effects on presynaptic AP-evoked Ca^2+^ influx were due to changes in intraboutonal free Ca^2+^ per channel opening, rather than in VGCC number or opening probability. While intraboutonal free Ca^2+^ represents an integrated measure of channel conductance, opening duration, and intraboutonal Ca^2+^ buffering capacity (Sabatini and Svoboda, 2000), involvement of the latter would be at odds with the VGCC subtype-specific nature of homeostatic effects; presynaptic homeostasis is therefore likely to be induced via direct modulation of P/Q-type currents. Several regulatory mechanisms have been reported for P/Q-type VGCC including association with SNAREs, G protein interactions, and channel binding by calmodulin (Catterall and Few, 2008), although these mechanisms predominantly regulate opening probability rather than channel currents. A more plausible suggestion comes from a recent study showing a role for alternative splicing of P/Q-type VGCC transcripts in various forms of presynaptic plasticity (Thalhammer et al., 2017), because alternative splicing specifically regulates unitary channel conductance (Chaudhuri et al., 2004).

We also examined presynaptic HSP at the level of neurotransmitter release, again finding a selective dependence on P/Q-type VGCC. We went on to probe whether mechanisms other than altered Ca^2+^ currents might contribute to presynaptic homeostatic plasticity. While we found no differences in the physical coupling of VGCC to release sites, an important determinant of presynaptic strength that has been suggested as a possible mechanism of presynaptic homeostasis (Müller et al., 2011), we did find that homeostasis was associated with modulation of the size of the RRP. This is consistent with most studies of homeostatic plasticity at both mammalian central synapses (Moulder et al., 2006, Müller et al., 2012, Murthy et al., 2001) and peripheral synapses (Davis and Müller, 2015), although some report no change (Zhao et al., 2011), suggesting that recruitment of RRP regulatory mechanisms may depend on synaptic maturity or homeostasis induction protocol, both of which differ among these various studies. An alternative explanation may be that effective RRP size, defined as those release-ready vesicles that can be released by AP stimulation, is controlled by presynaptic Ca^2+^ influx (Thanawala and Regehr, 2013). This might also explain why homeostatic changes in RRP size do not influence release in the presence of ω-agatoxin IVA in our experiments (Figure 3C), because Ca^2+^ influx through N-type VGCC is unchanged.

We asked whether changes in the size of both the RRP and the total recycling pool (TRP), which has been shown to increase in size following chronic silencing of network activity (Kim and Ryan, 2010), might also be mediated via a specific VGCC subtype. First, we established that the TRP is bidirectionally regulated during HSP, which confirms and extends the findings of Kim and Ryan (2010). We then showed that homeostatic changes in the size of both the RRP and TRP are mediated specifically via P/Q-type VGCC. In these experiments, it was also evident that Ca^2+^ influx through either channel type was able to release only a fraction of the TRP after which further stimulation was unable to release the remaining TRP vesicles, corresponding to a plateau of the SypH 2x signal. This interesting set of observations suggests that synaptic vesicles may be somehow committed to one VGCC subtype and cannot be released by Ca^2+^ entering through other VGCC subtypes. This might partly reflect the existence of active zone Ca^2+^ microdomains even during prolonged stimulation, such that the Ca^2+^ concentration required to drive vesicular membrane fusion is only reached in the immediate vicinity of VGCC (Parekh, 2008); vesicles docked to VGCC under blockade would therefore not be released. However, because the RRP of docked, release-competent vesicles is a relatively small fraction of the overall TRP (Rey et al., 2015), this would not explain the large discrepancy between TRP size with and without a VGCC blocker. Therefore, it is likely that the recruitment of new releasable vesicles to the active zone during a stimulus train is also channel-specific. It might be that the presynaptic vesicle trafficking machinery actively directs reserve vesicles toward Ca^2+^ microdomains associated with one or the other VGCC subtype; alternatively, synaptic vesicles themselves may have a VGCC-specific identity, which would add an intriguing dimension to the emerging theme of vesicle pool-specific molecular identities (Crawford and Kavalali, 2015).

All of the mechanistic changes we identified supporting HSP did so bidirectionally in response to either elevated or depressed network activity. However, a recent study at the *Drosophila* identifies several single gene mutations that block homeostatic presynaptic potentiation while leaving depression intact, suggesting that in this system at least these two responses show a degree of mechanistic independence (Gaviño et al., 2015). Despite this, they seem to converge on key elements such as *cacophony*-encoded VGCC (Gaviño et al., 2015), and while there may also be differences between mechanisms of homeostatic potentiation and depression at mammalian synapses, our data show that the involvement of P/Q-type VGCC is likewise conserved among both responses.

Our findings, together with work at the *Drosophila* NMJ (Davis and Müller, 2015), show that P/Q-type VGCC play an evolutionarily conserved role in mediating HSP at both invertebrate and mammalian synapses. P/Q-type VGCC are the sole driver of evoked release at invertebrate synapses, but at mammalian synapses this function is shared with N-type VGCC, which had previously been thought most likely to regulate presynaptic HSP (Frank, 2014, Kim and Ryan, 2013). It may therefore seem surprising that our data show that chronic modulation of basal release efficacy in mammals remains the sole responsibility of P/Q-type channels, however, it would be in keeping with the available functional data. Ca^2+^ influx through P/Q-type VGCC is more tightly coupled to release sites than that through N-type channels (Fedchyshyn and Wang, 2005, Ladera et al., 2009), and only P/Q-type channels support activity-dependent facilitation of Ca^2+^ currents (Inchauspe et al., 2004). Both of these observations suggest that P/Q-type VGCC might be better suited to mediating fast information transfer with high fidelity. N-type VGCC are, however, required for presynaptic Hebbian plasticity at some excitatory hippocampal synapses (Ahmed and Siegelbaum, 2009), while Hebbian plasticity specifically at the Schaffer collateral synapse seems to involve changes in RRP size rather than in presynaptic Ca^2+^ influx (Stanton et al., 2005, Wu and Saggau, 1994), underlining the multidimensional nature of presynaptic regulation. Together with our data, these observations suggest that the mechanisms of presynaptic Hebbian plasticity may be orthogonal to those of HSP, which could allow for effective homeostatic regulation of basal neurotransmission across synapses while preserving the relative differences in synaptic strength brought about by Hebbian changes.

Mutations in the pore-forming unit of the P/Q-type VGCC (CACNA1A) in humans give rise to the channelopathies familial hemiplegic migraine type 1 (FHM1) and episodic ataxia type 2 (EA2) (Cain and Snutch, 2011). The key phenotypes associated with these disorders, migraine, episodic ataxia, and epilepsy, are defined by long periods of neuronal stability punctuated by acute, relatively brief periods of dysfunction. It has been speculated that this reflects a disorder in HSP, because the process functions to prevent such departures from stable circuit function (Frank, 2014, Wondolowski and Dickman, 2013). Our work offers experimental support for this suggestion, because we show that effective regulation of P/Q-mediated Ca^2+^ currents, significantly impaired in mutated channels (Pietrobon, 2010), is essential for HSP. These findings therefore provide a firm basis for future work on the mechanistic basis of P/Q-type Ca^2+^ channelopathies and could also provide important insights into the pathogenesis of more common forms of migraine and epilepsy.

## Experimental Procedures

### Live Cell Imaging

All animal work was carried out in accordance with the Animals (Scientific Procedures) Act, 1986 (UK) and under project and personal licenses approved by the Home Office (UK). Experiments were performed on dissociated hippocampal cultures at days in vitro (DIV) 14–18 when synapses are mature. Before imaging, coverslips were washed in Tyrode’s buffer for 20 min to remove drug treatments. Experiments were carried out at room temperature in Tyrode’s buffer with 10 μM NBQX and 50 μM APV added to block recurrent activity. NH_4_Cl applications were done with 50 mM NH_4_Cl in substitution of 50 mM of NaCl. For SypH 2x experiments, folimycin (10 nM) was diluted into the medium. For experiments in which VGCCs were blocked, cultured hippocampal neurons were incubated with the P/Q-type channel blocker ω-agatoxin IVA (400 nM) or ω-conotoxin GVIA (400 nM) for at least 3 min prior to imaging. For more detail see the Supplemental Experimental Procedures.

### Image Analysis

All visible, stable varicosities within the image field were selected for analysis using a 2 μM diameter region of interest (ROI) and analyzed in ImageJ (https://imagej.nih.gov/ij) using the Time Series Analyzer plugin (https://imagej.nih.gov/ij/plugins/time-series.html). Data exported from ImageJ were background adjusted and, for pHluorins, normalized to the peak signal obtained following NH_4_Cl application (mean value of plateau over 5 s). Terminals were excluded from analysis if their peak response to stimulation was <2 (Ca^2+^ experiments and pHluorin experiments assessing vesicle pool sizes) or 4 (low frequency stimulation pHluorin protocol) SDs of baseline noise. Peak fluorescence in all experiments was taken at the end of the stimulation period. In experiments with SyGCaMP5, Ca^2+^ responses were averaged over 5 trials for each bouton. Analysis was performed in Microsoft Excel using custom-written macros.

### Statistical Analysis

Unless otherwise stated, the two-tailed unpaired Student’s t test was used to determine the statistical significance of observed differences between various conditions. p values >0.05 were regarded as non-significant. For more detail see the Supplemental Experimental Procedures.

## Author Contributions

A.F.J. designed the experiments, performed experiments, analyzed data, and wrote the manuscript. F.C.v.H., B.A.-M., and Z.P. performed experiments and analyzed data. N.J.E. wrote the manuscript.

## Supplemental Information

Document S1. Supplemental Experimental Procedures and Figures S1–S2

Document S2. Article plus Supplemental Information
