# Supplementary material for: Homeostatic Presynaptic Plasticity Is Specifically Regulated by P/Q-type Ca2+ Channels at Mammalian Hippocampal Synapses
Source: Cell Rep. 2017 Oct 10;21(2):341–50. doi: 10.1016/j.celrep.2017.09.061 (PMC5643522; doi:10.1016/j.celrep.2017.09.061)
Supplement: Document S1. Supplemental Experimental Procedures and Figures S1–S2 [file mmc1.pdf]

Cell Reports, Volume *21*

## Supplemental Information

# Homeostatic Presynaptic Plasticity Is Specifically Regulated by P/Q-type $Ca^{2+}$ Channels at Mammalian Hippocampal Synapses

**Alexander F. Jeans, Fran C. van Heusden, Bashayer Al-Mubarak, Zahid Padamsey, and Nigel J. Emptage**

## Supplemental figures

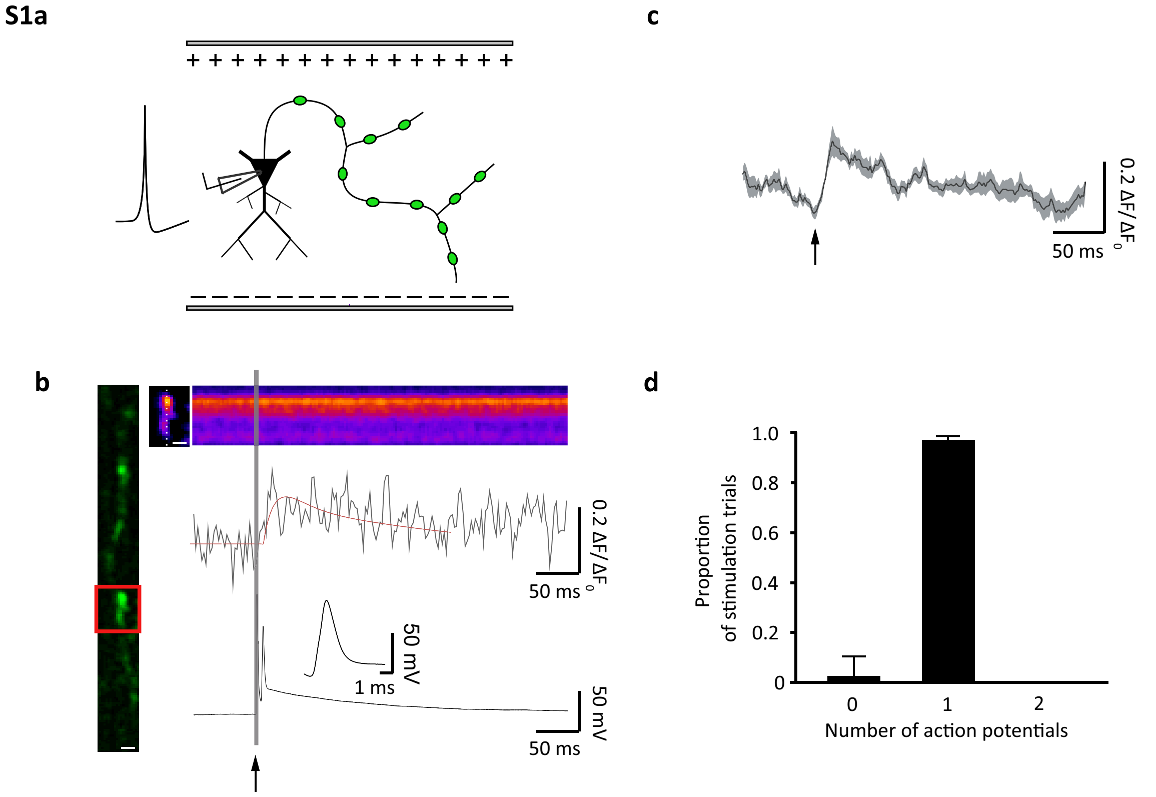


**Figure S1.** Field stimulation evokes single action potentials to drive detectable presynaptic $Ca^{2+}$ influx. Related to Figure 1. (**a**) Experimental configuration: dissociated hippocampal neurons expressing SyGCaMP5 were patched, and both electrophysiology and boutonal $Ca^{2+}$ fluorescence were monitored during low frequency (0.2 Hz) field stimulation. (**b**) Representative experiment. Left: SyGCaMP expressing boutons from a recorded neuron (image scale bar = 1 μm). Confocal laser scanning was restricted to a line across a single bouton (red box) to enable fast resolution imaging (500 Hz). Right: Sample line scan and quantified raw fluorescent changes are shown, along with time-locked electrophysiological recordings. The arrow and grey vertical line denote the time at which a single field stimulus was delivered. The stimulus evoked a single action potential, which is shown on two time scales, and a corresponding fluorescent increase in the bouton. An exponential function (red trace) has been fitted to the raw fluorescent trace to aid visualization. (**c**) Average field stimulation-evoked boutonal $Ca^{2+}$ transient, from 19 imaged boutons across 6 recorded cells. Shaded region represents ± s.e.m. (**d**) Quantification of action potential number evoked by single field stimuli (140 trials across 6 recorded cells). The vast majority of field stimuli (97%) resulted in single action potentials. Error bars represent ± s.e.m.

**S2**

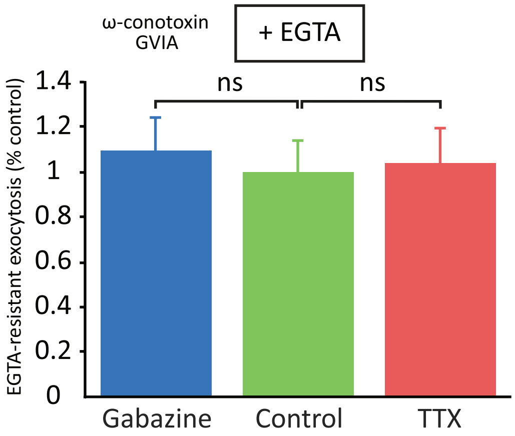


**Figure S2.** HSP does not elicit changes in the physical coupling of VGCC and release sites. Related to Figure 4. SypH 2x-expressing neurons were loaded with the cell-permeable $Ca^{2+}$ chelator EGTA-AM (200μm for 60s), then stimulated with 100AP at low frequency in the presence of folimycin and ω-conotoxin GVIA to isolate P/Q-type VGCC. We used incubation conditions for EGTA which led to a reduction of the response to 68.6% ± 0.017 (n = 185 synapses from 5 neurons) of its initial value under control conditions. Because of its relatively slow $Ca^{2+}$ binding rate, EGTA is expected to preferentially affect the release of vesicles that are located further from sites of $Ca^{2+}$ influx (Parekh, 2008); therefore, if homeostatic changes in release are associated with alterations in coupling of vesicles to VGCC, this should be reflected by changes in the sensitivity of release to EGTA. However, we found that neither gabazine nor TTX treatment changed the EGTA sensitivity of release compared to untreated controls, indicating that changes in the physical proximity of releasable vesicles and VGCC do not contribute to homeostatic regulation of release. Graph shows resistance to EGTA treatment expressed as % of control (-EGTA) response: gabazine-treated, 1.095 ± 0.144 (n = 191 synapses from 5 neurons), vehicle-treated control, 1 ± 0.137 (n = 185 synapses from 5 neurons); TTX-treated, 1.040 ± 0.156 (n = 112 synapses from 5 neurons). Error bars represent ± s.e.m. ns = non-significant.

## Supplemental experimental procedures

### Neuronal cultures and transfection

Hippocampal neurons from postnatal day 1 (P1) Wistar rats were seeded onto poly-D-lysine-coated coverslips and cultured in Neurobasal medium supplemented with 2% fetal calf serum (FCS), 2% B27, 1% Glutamax and 1% penicillin/streptomycin. The day after plating, half the medium was changed for Neurobasal supplemented with 2% B27 and 1% Glutamax only; this medium was used for all further feeds. Cells were transfected at 8 days *in vitro* (DIV) with either SypH 2x (gift of Dr. Y. Zhu) or SyGCamP5 (gift of Professor L. Lagnado) plasmids using Lipofectamine 2000 (Invitrogen). To induce chronic alterations in network activity, TTX (500nM) or gabazine (10μM) was added to the culture medium 24-36 hours before imaging.

### Live cell imaging

Experiments were performed on dissociated hippocampal cultures at DIV 14-18 when synapses are mature. Before imaging, coverslips were washed in Tyrode's solution (120mM NaCl, 2.5mM KCl, 20mM HEPES, 30mM glucose, 2mM $CaCl_2$, 2 mM $MgCl_2$, pH. 7.4) for 20 minutes to remove drug treatments. Coverslips were then mounted in a Chamlide EC-B18 stimulation chamber (Live Cell Instrument) on the stage of an Olympus IX-71 inverted microscope fitted with a 100X, NA 1.40 UPlanSApo objective and an Andor iXon EM CCD camera. Fluorescence illumination was supplied by a 100W mercury lamp used with appropriate neutral density filters and shuttered (Uniblitz CS25, Vincent Associates) during all non-data acquisition periods. Suitable fields were selected using the relatively good resting fluorescence signal exhibited by both pHluorin and GCaMP probes, the only criterion being that the boutons show normal, healthy morphology. APs were evoked by passing 20V, 1ms current pulses from a custom-made stimulation box via platinum electrodes. PHluorin imaging was carried out at 1Hz; $Ca^{2+}$ imaging was carried out at 10Hz. Stimulation, image acquisition and shuttering were all under the co-

ordinated control of WinWCP software (Strathclyde Electrophysiology Software). Experiments were carried out at room temperature in Tyrode's buffer. 10μM NBQX and 50μM APV were added to block recurrent activity. $NH_4Cl$ applications were done with 50mM $NH_4Cl$ in substitution of 50mM of NaCl. For SypH 2x experiments, folimycin (10nM) was diluted into the medium. For experiments in which VGCCs were blocked, cultured hippocampal neurons were incubated with the P/Q-type channel blocker ω-agatoxin IVA (400nM) or ω-conotoxin GVIA (400nM) for at least 3 min. prior to imaging.

**Combined patch clamping and live cell imaging**

To confirm that our stimulation protocol reliably elicits single AP, and that these drive presynaptic $Ca^{2+}$ influx which generates a detectable SyGCaMP5 signal, we recorded from dissociated neuronal cultures expressing SyGCaMP5 whilst simultaneously imaging presynaptic $Ca^{2+}$ responses. These recordings were carried out in whole-cell patch using patch electrodes (4-8MΩ) filled with internal solution (in mM: 135 KGluconate, 10 KCl, 10 HEPES, 2 $MgCl_2$, 2 $Na_2ATP$ and 0.4 $Na_3GTP$). Electrophysiological data was acquired with an Axoclamp 2A amplifier (Axon Insturments), recorded with WinWCP (Strathclyde Electrophysiology Software) and analysed with Clampfit (Axon Insturments) and Excel (Microsoft). Data was acquired at 3 kHz and sampled at 10 kHz. Images were acquired using a BioRad MRC-1000 confocal laser scanning system, equipped with a 488nm argon line, and LaserSharp software. Images were acquired through a 60x water-immersion objective (Olympus; 0.9 NA). Line scans were taken at 500Hz. Fluorescent changes were quantified as $\Delta F/F= (F - F_{baseline}/ F_{baseline} - F_{background})$ using ImageJ and Excel.

**Optical fluctuation analysis**

Optical fluctuation analysis allows the source of trial-to-trial variation in $Ca^{2+}$ responses to be identified (Sabatini and Svoboda, 2000). For each bouton, the coefficient of variation (CV = standard deviation/mean) of the peak amplitude of response over 5 trials was calculated. CV is related to the number of functional VGCC in each bouton, N, and their probability of opening in response to an AP, p, as given by: $CV^{-2} = (pN)/(1 - p)$. We calculated the mean $CV^{-2}$ across boutons for each of the three treatment conditions and plotted this against mean peak response. We then derived curves showing changes in $CV^{-2}$ predicted if homeostatic changes in $Ca^{2+}$ response amplitude were explained solely by changes in N, p or q, the free $Ca^{2+}$ concentration per channel opening. The estimate of p required for these predictions was taken from previous work (Ermolyuk et al., 2013). Curve fitting was carried out using linear regression and a t-test with correction for multiple comparisons was used to assess whether the experimental data differed significantly from that predicted by N, p or q changes.

**Statistical analysis**

Statistical analysis was performed using GraphPad Prism and MATLAB. Unless otherwise stated, the two-tailed unpaired Student's t-test was used to determine the statistical significance of observed differences between various conditions. Where other tests were used, this is clearly stated in the caption of the appropriate figure. P values greater than 0.05 were regarded as non-significant. Quantal analysis of pHluorin signals (Figure 1e) was performed in MATLAB by fitting a sum of multiple Gaussian distributions to the quantal frequency distribution. For this analysis, negative values of $\Delta F_{1AP}/\Delta F_{NH4Cl}$ and values >0.04 were excluded. The Gaussian mixture distribution model with the lowest AIC value was taken as the best fit.

## References

Ermolyuk, Y.S., Alder, F.G., Surges, R., Pavlov, I.Y., Timofeeva, Y., Kullmann, D.M., and Volynski, K.E. (2013). Differential triggering of spontaneous glutamate release by P/Q-, N- and R-type Ca2+ channels. Nature Neuroscience *16*, 1754-1763.

Sabatini, B.L., and Svoboda, K. (2000). Analysis of calcium channels in single spines using optical fluctuation analysis. Nature *408*, 589-593.
